# A one‐carbon path for fixing CO_2_: C_1_ compounds, produced by chemical catalysis and upgraded via microbial fermentation, could become key intermediates in the valorization of CO_2_ into commodity chemicals

**DOI:** 10.15252/embr.202050273

**Published:** 2020-03-29

**Authors:** Ari Satanowski, Arren Bar‐Even

**Affiliations:** ^1^ Max Planck Institute of Molecular Plant Physiology Potsdam Germany

**Keywords:** Synthetic Biology & Biotechnology, S&S: Economics & Business, Metabolism

## Abstract

Chemicals synthesized directly from CO_2_ are a sustainable alternative to fossil fuels. Increasing efficiency and specificity will require a combination of chemical and biological processes.

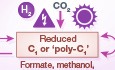

Our society relies on enormous amounts of chemical products—plastics, medicines, textiles, construction materials, flavors, and so on—most of which are synthesized from fossil resources, mainly oil. With growing concerns about global climate change and increasing demand for sustainable production schemes, it becomes clear that we need to wean ourselves from our dependency on fossil carbons. Ultimately, the only truly sustainable feedstocks for a circular carbon economy are CO_2_ and renewable energy 1: The first supplies the elemental carbon, while the second provides the energy for converting CO_2_ into useful products. Such a circular, sustainable carbon economy has great potential for sequestering carbon from the atmosphere and thus, at least partially, counteracting the continuous release of greenhouse gases by human activities and the effects of global warming.

With growing concerns about global climate change and increasing demand for sustainable production schemes, it becomes clear that we need to wean ourselves from our dependency on fossil carbons.

## CO_2_ valorization routes: the prospect of coupling abiotic and biotic catalyses

**Figure 1:**
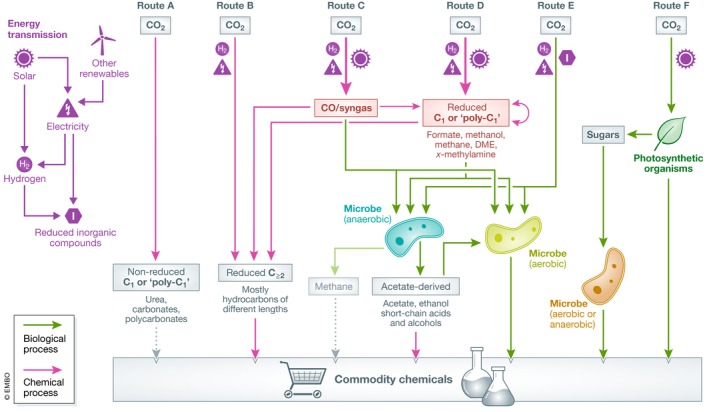
Alternative routes for CO_2_ conversion into commodity chemicals Valorization of CO_2_ can proceed by different routes, utilizing different sources of energy (purple icons) and involving different carbon intermediates. Chemical (abiotic) processes are shown by orange arrows while biological processes are represented by green arrows. Schematic conversion between energy sources is shown on the left of the figure.

There is a plethora of methods to convert CO_2_ into value‐added chemicals 2, 3, 4, 5, 6. These routes are usually classified based on the catalytic approach. Instead, we find it useful to discuss these methods according to a different classification that focuses on the carbon intermediates directly produced from CO_2_ (Fig 1). Following this perspective, we highlight the inherent difficulties and limitations that different CO_2_ conversion methods face, concluding that the coupling of chemical and biological processes is an attractive strategy. As the availability of renewable energy is a key limiting resource and a substantial factor of the production costs 1, we put particular emphasis on energy efficiency and product specificity—given that lower specificity wastes energy in synthesizing unwanted byproducts.

Non‐reductive CO_2_ valorization via chemical methods (route A in Fig 1) is the commercially most advanced route; it is used, for instance, for the annual global production of more than 200 million tons of urea 2. CO_2_ can also serve as a feedstock for the production of other carbonate derivatives such as dimethyl carbonate and polycarbonates 2, 4. These routes are already economically viable and have great potential for capturing significant amounts of CO_2_ from the atmosphere 2. However, non‐reductive methods for valorizing CO_2_ are limited to a small number of specific compounds, which cannot be easily converted to other commodity chemicals. Hence, it is imperative to explore other routes which can support the conversion of CO_2_ into a wider array of chemicals.

Direct reduction of CO_2_ into multi‐carbon (C_≥2_) compounds (route B in Fig 1) can be achieved using several chemical strategies: for example, catalytic hydrogenation such as Fischer–Tropsch, plasma technology, or electrochemistry 4, 5. While each of these methods has its own advantages and drawbacks, they all suffer from common problems including low product selectivity and limited product spectrum, which require extensive downstream chemical processing to produce different chemicals. Another drawback of the most advanced of these routes—catalytic hydrogenation technologies—is the extreme temperature and/or pressure needed; downsizing to a scale compatible with distributed CO_2_ sources is still a challenge 5. On the other hand, methods that operate under mild conditions and at a smaller scale, such as plasma and electrochemical technologies, produce C_≥2_ compounds only at low energetic efficiency and at a low rate 3, 4, 5, 6.

… it is imperative to explore other routes which can support the conversion of CO_2_ into a wider array of chemicals.

Alternatively, CO_2_ can be reduced to the C_1_ intermediate carbon monoxide (CO)—or to syngas, a mixture of CO and hydrogen (route C in Fig 1)—using diverse chemical strategies, such as reverse water‐gas shift, plasma technology, electrochemistry, and photochemistry 3, 4, 5, 6. Unlike the synthesis of C_≥2_ compounds, CO and syngas can be produced at very high specificity (close to 100%) and energetic efficiency. Syngas can then be efficiently converted to other reduced C_1_ compounds, such as methanol. Alternatively, CO_2_ can be directly reduced to C_1_ compounds other than CO (route D in Fig 1), including formic acid, methane and methanol by electrochemistry, photochemistry, plasma technology, and catalytic hydrogenation 3, 4, 5, 6. The production of these C_1_ intermediates achieves somewhat lower specificity and energetic efficiency than CO/syngas production, but it is still substantially higher than direct reduction of CO_2_ to C_≥2_ compounds.

Both syngas and the other reduced C_1_ compounds can be chemically upgraded to C_≥2_ compounds via catalytic hydrogenation, such as Fischer–Tropsch, methanol‐to‐gasoline, methanol‐to‐olefin, or methanol‐to‐aromatics processes 5. While some of these production methods are commercially established on an industrial scale, they still require extreme conditions and are sensitive to impurities in the feedstock. Moreover, these processes generate a non‐specific mixture of compounds that requires extensive processing before it can be further used to synthesize commodity chemicals.

## Biological processes for CO_2_ valorization

Taken together, chemical methods excel in reducing CO_2_ into C_1_ compounds with high specificity and energetic efficiency. Some of the emerging CO_2_ reduction methods—especially electrochemistry—can also operate under ambient conditions and at a flexible scale. In contrast, the production of C_≥2_ compounds from CO_2_ requires extreme reaction conditions, suffers from low product specificity, and involves considerable downstream processing. A growing bio‐manufacturing industry now aims to develop solutions to these issues, as biological systems operate at mild conditions, excel at product specificity, and can achieve economic viability at a smaller scale in decentralized production facilities 7.

…diverting agricultural resources towards bio‐production is not a sustainable long‐term solution: it directly competes with food production and threatens biodiversity if more natural areas are converted for agricultural production.

The industrially most established biological processes for CO_2_ valorization rely on photosynthesis (route F in Fig 1). Sugars or other carbohydrates produced by agricultural crops are further metabolized by microorganisms to produce compounds of interest. Alternatively, plants or algae can be used directly, for example, to produce fatty acids or oil from plant seeds. However, diverting agricultural resources toward bio‐production is not a sustainable long‐term solution: It directly competes with food production and threatens biodiversity if more natural areas are converted for agricultural production. Using non‐consumable biomass, such as lignocellulose and algae, would avoid competing with food production, but these materials are difficult to process. Above all, photosynthesis is very inefficient energetically and typically stores less than 1% of sunlight energy in chemicals 8.

Rather than relying on plants to assimilate CO_2_, a relatively new approach to generate commodity chemicals explores chemolithotrophic microorganisms that are able to reduce CO_2_ using energy sources other than light (route E) 8. One strategy for chemolithotrophic CO_2_ assimilation is to directly transfer electrons from an electrode to a microorganism. This approach has a high energetic efficiency, but it is limited by a very low current density, and therefore requires a very large electrode surface area which dramatically increases capital expenditure 9. Inorganic electron donors—such as ferrous iron or nitrogen‐ and sulfur‐containing chemicals—can be regenerated electrochemically, but their consumption by microorganisms is limited to low energetic efficiencies, typically < 20% 9. The exception is hydrogen, a low reduction potential compound, which can be metabolized at a high energetic efficiency by anaerobic organisms such as acetogens and methanogens 9. Yet, the low solubility of hydrogen (> 40‐fold lower than CO_2_) limits mass transfer to the culture and thus productivity, requires high pressure for operation, and further necessitates recycling of feedstock from the bioreactor off‐gas.

Such hybrid abiotic/biotic C_1_‐based production chains have the potential to operate at high specificity and energetic efficiency under ambient conditions…

Alternatively, reduced C_1_ compounds from chemical synthesis could serve as microbial feedstocks to simultaneously provide a source for both carbon and energy 9. This strategy integrates abiotic and biotic catalyses and harnesses their respective advantages while bypassing their drawbacks: CO_2_ is activated by chemical means to produce a C_1_ compound at high specificity and efficiency (routes C and D), while microorganisms utilize the C_1_ intermediate to produce specific C_≥2_ compounds. Such hybrid abiotic/biotic C_1_‐based production chains have the potential to operate at high specificity and energetic efficiency under ambient conditions, and are therefore an attractive option for a future circular carbon economy.

## Benefits of CO_2_ valorization via C_1_ compounds

One of the main advantages of such a hybrid C_1_‐based production chain is its immense flexibility in terms of renewable energy sources and products. With regard to the former, C_1_ feedstocks can be produced via multiple routes, each with its own advantages and disadvantages. For example, CO and formic acid can be efficiently produced via electrochemical or photochemical reduction of CO_2_ 3, 4, 5, 6. While photochemical reduction bypasses costs and complexities that arise from the multi‐step processes of generating electricity and using an electrochemical apparatus, it is less flexible than electrochemistry which is not restricted to solar power and can rely on different energy sources.

**Figure 2:**
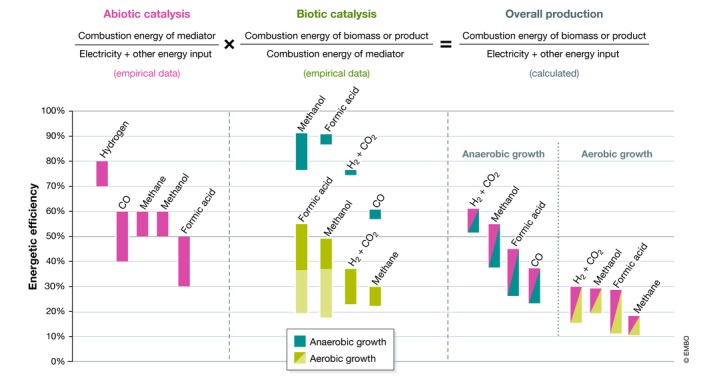
The energetic efficiency of C_1_‐based production chains with different C_1_ mediators Bars correspond to empirical ranges for abiotic production of several C_1_ compounds (and hydrogen as a reference) and for their biological valorization. The overall efficiency of the production chain (on the right) is calculated as a product of the two process efficiencies. Aerobic biological utilization (pink bars) of formic acid and methanol can proceed via different metabolic routes with varying efficiency; values from less efficient routes are shown in a brighter color, while values corresponding to the most efficient routes are shown in a dark color 9.

Electrochemical production of CO and formic acid can already be achieved at relatively high efficiencies of 40–60% (red bars in Fig 2). The production of renewable methane and methanol from hydrogenation of CO_2_—where hydrogen is produced from water electrolysis—is already performed at an industrial scale with overall energetic efficiencies of 50–60% (red bars in Fig 2) 3, 4, 5. While the efficiency of producing C_1_ feedstocks from CO_2_ is somewhat lower than that of hydrogen from water electrolysis, it can still surpass, or at least approach, 50%. In addition, some of the C_1_ feedstocks—mainly CO and syngas—can be recovered from waste streams, including industrial flue gases and gasification of organic wastes. As syngas can be converted to other C_1_ compounds, these can, in principle, be generated from waste streams.

The hybrid abiotic/biotic C_1_‐based approach also offers much more production flexibility, as microorganisms cultivated on C_1_ feedstocks could be engineered to produce a wide variety of carbon compounds. Here, it is important to distinguish between production under aerobic and anaerobic conditions. Only specific microorganisms, such as acetogens and methanogens, can grow anaerobically on C_1_ feedstocks such as CO, formic acid, and methanol without the need for electron acceptors other than CO_2_. Anaerobic growth supports a high energetic efficiency of 60–90% from feedstock to product (blue bars in Fig 2), such that the overall energetic efficiency from CO_2_ to product can reach 30–50% (right‐hand side of Fig 2) 9.

However, anaerobic bio‐production from C_1_ feedstocks is limited to a narrow product spectrum, since biosynthesis must be coupled to cellular energy conservation. The few chemicals that can be produced anaerobically are mostly derived from the metabolic intermediate acetyl coenzyme A. Nonetheless, anaerobic biosynthesis is already used on an industrial scale. For example, the production of ethanol from C_1_‐based waste gases (CO or syngas) by companies such as LanzaTech has reached a commercial stage. Similarly, Siemens and Evonik have developed an integrated process by which syngas, electrochemically produced from CO_2_ and water, is converted by a consortium of anaerobic bacteria into short‐chain fatty alcohols. Acetate, ethanol, and other direct products of these anaerobic biological processes can be further upgraded to complex molecules either chemically or biologically (Fig 1). An example for a chemical upgrading is the conversion of ethanol to jet fuel as pursued by LanzaTech and the US Energy Department's Pacific Northwest National Laboratory.

The hybrid abiotic/biotic C_1_‐based approach also offers much more production flexibility, as microorganisms cultivated on C_1_ feedstocks could be engineered to produce a wide variety of carbon compounds.

In contrast to anaerobes, aerobic microorganisms have a very broad product spectrum as their biosynthesis is not coupled to cellular energy conservation 9. The energetic efficiency of aerobic bio‐production highly depends on the desired product and the metabolic pathway used to support growth on the C_1_ feedstock (pink bars in Fig 2), but it is generally lower than that achieved by anaerobic microorganisms 9. Especially promising are formic acid and methanol, the biological assimilation of which can support an energetic efficiency of up to 50% under aerobic conditions, and an overall energetic efficiency—from CO_2_ to product—of up to 30%. In fact, among the different C_1_ feedstocks, formic acid and methanol support the highest energetic efficiency both under aerobic and anaerobic conditions 9. Formic acid has only recently been suggested as an industrial feedstock, and bio‐production based on this carbon source—mainly of alcohols and bioplastics—is rather limited and still not commercially mature. In contrast, methanol has been explored as a microbial feedstock for decades, mostly for producing proteins as feed or food supplement. While not commercial yet, the methanol‐based production of a wider array of products—including amino acids, polyamines, terpenoids, and organic acids—has been extensively studied.

**Figure 3:**
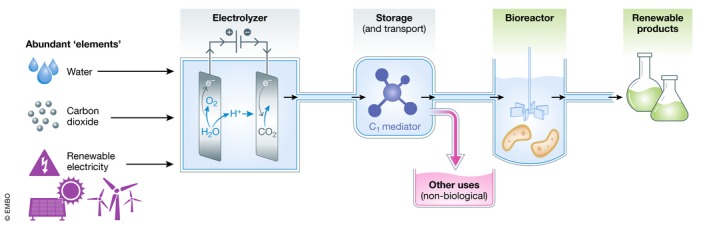
The C_1_‐based production chain can achieve high flexibility by decoupling the chemical and biological processes The C_1_‐mediator is temporarily stored or even transported, and can be used for other applications as well.

Another key advantage of the C_1_‐based production chain is its modularity, as individual steps in the production chain can be decoupled from each other, which makes the entire process more flexible and robust 9. Specifically, the C_1_ mediator produced from CO_2_ can be stored and transported, which enables spatial and temporal decoupling of the chemical and biological processes (Fig 3). This ensures that the operational conditions of each process can be optimized separately and that the intrinsic mismatch between the production rates of the chemical and biological processes does not hamper productivity 9. Moreover, while the viability and productivity of a microbial culture relies on continuous feedstock supply, the availability of renewable energy for synthesizing the feedstock can dramatically fluctuate over short time scales. This makes temporal decoupling of the chemical and biological processes vital to ensure the robustness and stability of the production chain.

C_1_ intermediates add further flexibility as they can be diverted to other, non‐biological uses (brown arrow in Fig 3). For example, the electrochemical reduction of CO_2_ to formic acid is being actively pursued as a method for storing excess electricity that can be released when needed in formic acid fuel cells. Hence, according to the needs, electrochemically produced formic acid can be used either for electricity regeneration or for bio‐production of commodity chemicals. Similarly, renewably produced methane can be integrated into the existing natural gas infrastructure or fed to microbes to produce chemicals.

## Challenges for integrating abiotic and biotic processes

Combining abiotic and biotic processes can be a difficult task. One major challenge is the use of the gaseous C_1_ feedstocks in aqueous fermentation. Just as hydrogen, CO has a very low solubility in water, which limits the rate of feedstock delivery to the microbial culture and requires energy‐intensive pressurization in order to obtain reasonable productivities. Methane has a somewhat higher solubility, but its mass transfer can still be a limiting factor for production. Moreover, gaseous feedstocks tend to escape via the exhaust gas before they are completely utilized, which requires costly recycling. Novel bioreactor designs and optimized cultivation conditions could at least partially address this problem, but they usually come with increased capital expenditure or operating expenses.

A seemingly simpler solution is to use miscible C_1_ intermediates, namely formic acid and methanol. Moreover, these liquid feedstocks can be easily stored and transported. Yet, the use of formic acid and methanol comes with its own challenges. First, aqueous solutions of C_1_ compounds generated by abiotic processes usually contain impurities, some of which originate from the CO_2_ source itself while others stem from the production process (for instance, electrolytes from the electrolyzer). While microorganisms are generally much more tolerant to such impurities than chemical processes 7, they can still be severely inhibited by specific molecules. Removing such impurities from a solution of formic acid or methanol could be quite costly as separating a compound from an aqueous solution is generally more expensive than from a gaseous mixture.

On a similar note, as the production of both formic acid and methanol results in a diluted product—since formic acid is produced in an aqueous solution and CO_2_ hydrogenation to methanol generates water—additional energy is required for concentrating the feedstock. Importantly, however, unlike chemical processes that could be inhibited by the presence of water, microorganisms can be fed with a diluted solution. Still, feeding with a diluted feedstock comes with a cost, as the extra water added to the bioreactor needs to be subsequently expelled in order to keep a constant volume. This enforces a continuous cultivation mode that could limit product titer. Here, it is vital to identify the right balance between minimizing the energy required for concentrating the feedstock and obtaining sufficiently high titers.

Another challenge is the counterproductive release of CO_2_ during the biological consumption of C_1_ feedstocks. First, since biomass and most products are more reduced than CO and formic acid, a substantial fraction of these feedstocks needs to be oxidized to CO_2_ in order to generate the reducing power for the metabolic conversion of the rest of the feedstock into larger molecules. The use of a more reduced feedstock, such as methanol, can minimize this CO_2_ release. Second, under aerobic conditions, a considerable amount of the substrate is oxidized to provide energy for cell growth and bio‐production, thus increasing the release of CO_2_. Operating under anaerobic conditions eliminates this carbon loss, but, as mentioned above, is limited to a few products. An interesting approach to minimize carbon loss is to recycle CO_2_ from the exhaust gas and feed it back to the abiotic process which produces the C_1_ feedstock. However, this recycling comes at a cost, as the transfer of CO_2_ from the biological to the chemical process will usually require spatial and temporal coupling of the two, thus limiting the overall flexibility of the production chain.

## The next steps

Compared to conventional chemical and biological industries, the technologies involved in the C_1_‐based production chain are at a lower level of maturity. Moreover, the production chain described here heavily relies on the integration of technologies from different fields. Hence, further development of a C_1_‐based economy will require interdisciplinary research. Advances in CO_2_ capture and purification, chemical catalysis, microbiology, synthetic biology, and bioprocess engineering will be key drivers for this progress. Fortunately, several of these research fields have already received attention in the context of other, more established industrial schemes. For example, CO_2_ capture and purification from industrial point sources and direct air capture of CO_2_ are intensely investigated. Similarly, chemical production of C_1_ compounds is being pursued for storing renewable energy. On the biological side, metabolic engineering of relevant microorganisms has already achieved efficient biosynthesis of various industrially relevant chemicals, including fuels, solvents, acids, and plastics 7.

… the production chain described here heavily relies on the integration of technologies from different fields.

Yet, some aspects of the hybrid abiotic/biotic C_1_‐based production chain require more dedicated research and innovation. One is the establishment of advanced microbial platforms for converting C_1_ substrates into commodity chemicals. While various groups of microorganisms are actively being researched, these are not necessarily ideal from an industrial perspective, owing to limitations in genetic tools and metabolic understanding or unfavorable cultivation conditions. Further study, engineering and optimization of natural C_1_‐utilizing organisms—such as acetogens, *Cupriavidus necator*,* Methylobacterium extorquens*, and *Bacillus methanolicus*—would be one approach, which is actively pursued by multiple research groups and several companies. Alternatively, model microorganisms which are extensively used industrially but cannot naturally grow on C_1_ feedstocks—for example, *Escherichia coli*,* Saccharomyces cerevisiae*, and *Corynebacterium glutamicum*—could be engineered to utilize these compounds. This strategy is less developed, but it might hold the potential to advance the field dramatically. Rather than relying on metabolic routes existing in nature, it allows the use of synthetic biology for rationally designing more efficient alternatives for biological conversion of C_1_ feedstocks 10.

As electricity is a principal feedstock of the production chain, its cost has a major effect on the overall economics.

While evaluating the detailed economics of C_1_‐based production chains is beyond the scope of this article, we put forward several aspects that need to be addressed for the technology to become economically viable and able to compete with fossil resources. As electricity is a principal feedstock of the production chain, its cost has a major effect on the overall economics. Fortunately, the cost of generating electricity from sunlight or wind keeps dropping: Several solar and onshore wind projects have electricity production costs of less than 4 cents per kWh 6. Moreover, C_1_‐based production provides an efficient strategy to valorize surplus electricity produced at off‐peak hours. Yet, eventually, carbon‐neutral electricity production would have to be vastly expanded to fully decouple the chemical industry from fossil resources: About half of the projected global electricity production in 2030 would be required to enable the production of all value‐added chemicals from CO_2_ 1.

Another major cost driver is capital expenditure for the electrochemical production of the C_1_ intermediates. Addressing this challenge would require decreasing the cost of electrodes by reducing the use of precious metals, such as anode iridium, and increasing cathodic current density preferably to ~ 1 A/cm^2^ 9. Purifying CO_2_ from point sources, such as the flue gas of a steel or cement factory, is relatively cheap, at € 30–50/ton CO_2_. In the long term, however, direct air capture of CO_2_ would be required to achieve a net removal from the atmosphere, but the cost of this process is currently an order of magnitude higher than using CO_2_ point sources. Subsidies for CO_2_ utilization and/or taxation on CO_2_ release could help make C_1_‐based technologies more competitive. Finally, unlike traditional fermentation, which has been extensively studied and optimized, biological conversion of C_1_ intermediates into chemicals is still in its infancy. Achieving high yields, titers, and productivities is essential for a C_1_‐based production chain to become economically viable.

To fully understand the environmental and economic impact of C_1_‐based production will also require rigorous life cycle and techno‐economic analyses. These analyses are not yet possible owing to the early stage of development of some of the required technologies and a lack of communication between chemists and biologists. Once stronger interdisciplinary interactions are established and more information is available, an accurate comparison between the C_1_‐based production chain and alternative CO_2_ utilization schemes would allow identifying the advantages and drawbacks of each. Ultimately, policy makers will rely on such data to make legislative decisions with regard to carbon pricing, subsidies for specific technologies, and allocation of research funds. Similarly, commercial stakeholders have strong incentives to explore novel routes for CO_2_ valorization given the societal demand for a low‐carbon emission industry and the growing market for sustainable products.

## Conflict of interest

A.B.‐E. is cofounder of b.fab, exploring the commercialization of microbial bio‐production using formate as feedstock. The company was not involved in any way in performing or funding this study.

## References

[1] Katelhon A , Meys R , Deutz S , Suh S , Bardow A (2019) Climate change mitigation potential of carbon capture and utilization in the chemical industry. Proc Natl Acad Sci USA 116: 11187–11194 31085651 10.1073/pnas.1821029116 PMC6561304

[2] Hepburn C , Adlen E , Beddington J , Carter EA , Fuss S , Mac Dowell N , Minx JC , Smith P , Williams CK (2019) The technological and economic prospects for CO_2_ utilization and removal. Nature 575: 87–97 31695213 10.1038/s41586-019-1681-6

[3] Snoeckx R , Bogaerts A (2017) Plasma technology ‐ a novel solution for CO_2_ conversion? Chem Soc Rev 46: 5805–5863 28825736 10.1039/c6cs00066e

[4] Artz J , Muller TE , Thenert K , Kleinekorte J , Meys R , Sternberg A , Bardow A , Leitner W (2018) Sustainable conversion of carbon dioxide: an integrated review of catalysis and life cycle assessment. Chem Rev 118: 434–504 29220170 10.1021/acs.chemrev.7b00435

[5] Grim RG , Huang Z , Guarnieri MT , Ferrell JR , Tao L , Schaidle JA (2020) Transforming the carbon economy: challenges and opportunities in the convergence of low‐cost electricity and reductive CO_2_ utilization. Energy Environ Sci 13: 472–494

[6] De Luna P , Hahn C , Higgins D , Jaffer SA , Jaramillo TF , Sargent EH (2019) What would it take for renewably powered electrosynthesis to displace petrochemical processes? Science 364: eaav3506 31023896 10.1126/science.aav3506

[7] Clomburg JM , Crumbley AM , Gonzalez R (2017) Industrial biomanufacturing: the future of chemical production. Science 355: eaag0804 10.1126/science.aag0804 28059717

[8] Claassens NJ , Sousa DZ , Dos Santos VA , de Vos WM , van der Oost J (2016) Harnessing the power of microbial autotrophy. Nat Rev Microbiol 14: 692–706 27665719 10.1038/nrmicro.2016.130

[9] Claassens NJ , Cotton CA , Kopljar D , Bar‐Even A (2019) Making quantitative sense of electromicrobial production. Nat Catal 2: 437

[10] Yishai O , Lindner SN , Gonzalez de la Cruz J , Tenenboim H , Bar‐Even A (2016) The formate bio‐economy. Curr Opin Chem Biol 35: 1–9 27459678 10.1016/j.cbpa.2016.07.005

